# 

**Published:** 1906-08-11

**Authors:** 


### Medical Vacancies.

Bradford, Royal Infirmary.—Medical Officer.

Brighton, Sussex County Hospital.—Third House Surgeon.

Brighton Throat and Ear Hospital, Church Street, Queen's Road.—Non-resident House Surgeon.

Cambridge University.—R. C. Brown Research Scholarship in Special Pathology.

Central London Throat and Ear Hospital, Gray's Inn Road. Registrar (Honorary).

Cheltenham General Hospital.—Junior House Surgeon.

Cheshire, Crossley Sanatorium, Delamere Forest.—Assistant Medical Officer.

Devonport, Royal Albert Hospital.—Assistant Resident Medical Officer.

Hounslow Hospital.—Honorary Ophthalmic Surgeon.

Liverpool Infectious Diseases Hospital.—Assistant Resident Medical Officer.

Manchester, St. Mary's Hospitals.—Six District Obstetric Officers.

Newcastle-on-Tyne Dispensary.—Visiting Medical Assistant.

Newport and Monmouthshire Hospital.—Junior Resident Medical Officer.

Oxford Radcliffe Infirmary and County Hospital.—House Physician, House Surgeon, and Junior House Surgeon.

Poplar Hospital for Accidents, Poplar, E.—Assistant House Surgeon.

St. Helens County Borough.—Assistant Medical Officer (female).

Sheffield Royal Hospital.—Assistant House Surgeon.

Southwark Union Infirmary, East Dulwich, S.E.—Second Assistant Medical Officer.

Stockport Infirmary.—Junior Assistant House Surgeon.

Stroud General Hospital.—House Surgeon.

Truro, Royal Cornwall Infirmary.—House Surgeon.

West Bromwich District Hospital.—Resident Assistant House Surgeon.

Worcester County and City Asylum.—Third Assistant Medical Officer.

Sir Edward Sassoon, Bart., M.P., will preside at a banquet in November next, in aid of the funds of St. John's Hospital for Diseases of the Skin, Leicester Square.

As the result of investigations extending over a period of many years, with the aid of expert assistance, Sir Almroth E. Wright, M.D., F.R.S., has written a book which Messrs. Archibald Constable will shortly publish, which cannot fail to have a marked effect on the results obtainable by all students using the microscope; hitherto this instrument has been all too commonly treated by rule of thumb, and without those considerations of the principles of microscopic technique which are so essential to the attainment of accurate and scientific results. The object of the book is to show how best to get from any class of instrument the highest degree of satisfaction in proportion to its range. The text of the whole book goes hand in hand with experiments. The work also contains a complete vocabulary of technical terms relating to the microscope.

### A Dozen Books every Nurse should Possess.

| | | |
|---|---|---|
| 1. | **A HANDBOOK FOR NURSES.**<br>By J. K. WATSON, M.D.<br>Profusely Illustrated. Cloth gilt. | 5/- net, 5/4 post free. |
| 2. | **A COMPLETE HANDBOOK OF MIDWIFERY.**<br>By J. K. WATSON, M.D.<br>Illustrated with 143 Diagrams. Cloth gilt. | 6/- net, 6/4 post free. |
| 3. | **THE NURSES' DICTIONARY OF MEDICAL TERMS AND NURSING TREATMENT.**<br>Compiled by HONNOR MORTEN.<br>Fourth Fdition. Suitable size for the apron pocket. Cloth. | 2/- post free. |
| 4. | **PRACTICAL GUIDE TO SURGICAL BANDAGING AND DRESSINGS.**<br>By WM. JOHNSON SMITH, F.R.C.S.<br>Suitable size for the apron pocket. Profusely Illustrated. Cloth. | 2/- post free. |
| 5. | **SURGICAL INSTRUMENTS AND APPLIANCES USED IN OPERATIONS.**<br>By HAROLD BURROWS, F.R.C.S.<br>A Classified and Illustrated List and Explanatory Notes. Cloth. | 1/6 net, 1/8 post free. |
| 6. | **NURSING HINTS TO PROBATIONERS ON PRACTICAL WORK.**<br>By MARY ANNESLEY VOYSEY.<br>Cloth. | 2/- net, 2/3 post free. |
| 7. | **ELEMENTARY ANATOMY AND SURGERY FOR NURSES.**<br>By WM. McADAM ECCLES, M.S., M.B.<br>Profusely Illustrated. Cloth. | 2/6 post free. |
| 8. | **ELEMENTARY PHYSIOLOGY FOR NURSES.**<br>By C. F. MARSHALL, M.D., B.Sc., F.R.C.S.<br>Profusely Illustrated. Cloth. | 2/- post free. |
| 9. | **SURGICAL WARD-WORK AND NURSING.**<br>By ALEXANDER MILES, M.D.Edin.<br>Second Edition. Illustrated. Cloth gilt. | 3/6 net, 3/10 post free. |
| 10. | **OPHTHALMIC NURSING.**<br>By SYDNEY STEPHENSON, M.B., F.R.C.S.E.<br>New Revised Edition. Illustrated. | 3/6 net, 3/10 post free. |
| 11. | **THE EXAMINATION OF THE URINE.**<br>By J. K. WATSON, M.D.<br>Cloth boards. Suitable size for the apron pocket. | 1/- net, 1/1 post free. |
| 12. | **LECTURES ON NURSING OF INFECTIOUS DISEASES.**<br>By Dr. J. WOOLLACOTT.<br>Cloth. | 2/6 net, 2/9 post free. |

**SEND FOR LATEST CATALOGUE OF NURSING MANUALS.**

*The above Books are obtainable of all Booksellers, or of*

